# Supplementary material for: LPMO-oxidized cellulose oligosaccharides evoke immunity in Arabidopsis conferring resistance towards necrotrophic fungus B. cinerea
Source: Commun Biol. 2021 Jun 11;4:727. doi: 10.1038/s42003-021-02226-7 (PMC8196058; doi:10.1038/s42003-021-02226-7)
Supplement: Supplementary file 1 — Supplementary Material [file 42003_2021_2226_MOESM1_ESM.pdf]

# SUPPLEMENTARY Information for:

**LPMO-oxidized cellulose oligosaccharides evoke immunity in Arabidopsis conferring resistance towards necrotrophic fungus *B. cinerea***

**Marco Zarattini[1] , Massimiliano Corso[2] , Marco Antonio Kadowaki[1] , Antonielle Monclaro[1], Silvia Magri[1] , Irma Milanese[1] , Sylvie Jolivet[2] , Mariana Ortiz de Godoy[1] , Christian Hermans[3] , Mathilde Fagard[2] and David Cannella[1 *]**

[*] corresponding author and material availability at: david.cannella@ulb.ac.be

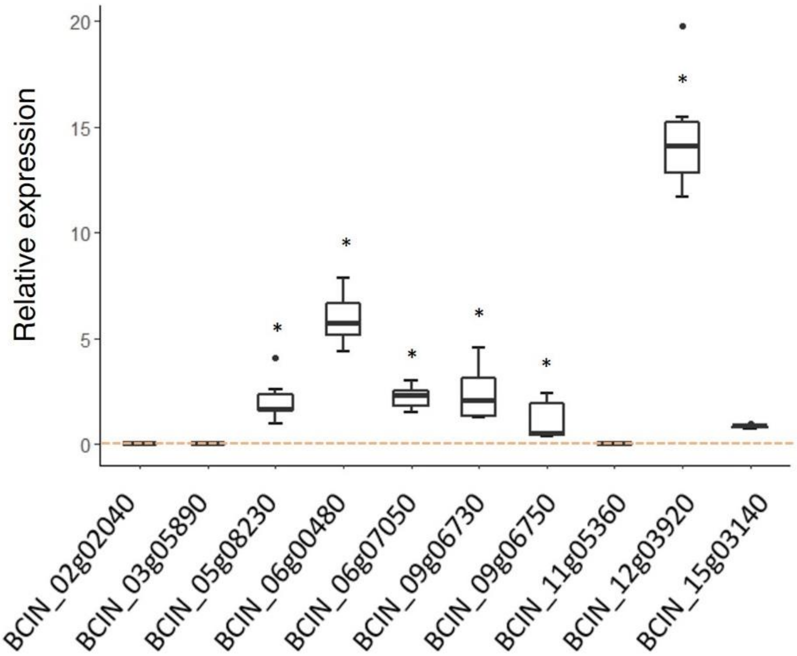


**Supplementary Figure 1: gene expression analysis for *AA9* gene family in *B. cinerea* during infection on Arabidopsis.** The *AA9*s gene expression was evaluated during Arabidopsis-*B. cinerea* interaction. Three independent pools of fifteen five-week-old Arabidopsis leaves (n = 3) were drop-treated with the *B. cinerea* spores ($10^5$ spores $ml^{-1}$) and gene expression was evaluated 48 h post-infection by using the *BcTUB* as reference gene. Median values are plotted in the boxes with data generated from three independent experiment. Relative expression levels of each *AA9* genes were evaluated also in *B. cinerea* growth on PDA solid medium (indicated by orange dotted lines). *BcTUB* was used as reference gene. Asterisks indicate significant differences according to one-way ANOVA test ($p < 0.05$).

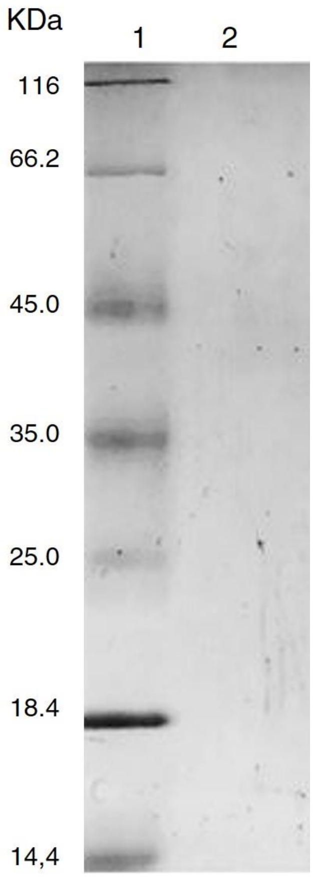


**Supplementary Figure 2: SDS-page analysis of AA9_COS**. The SDS-page electrophoretic run was performed on 100 µM AA9_COS to evaluate the presence of LPMO. The gel reveals the presence of no proteins in the solution. Line 1: EZ-RUNTM Protein Marker (Fisher Scientific, USA), Line 2: 100 µM AA9_COS.

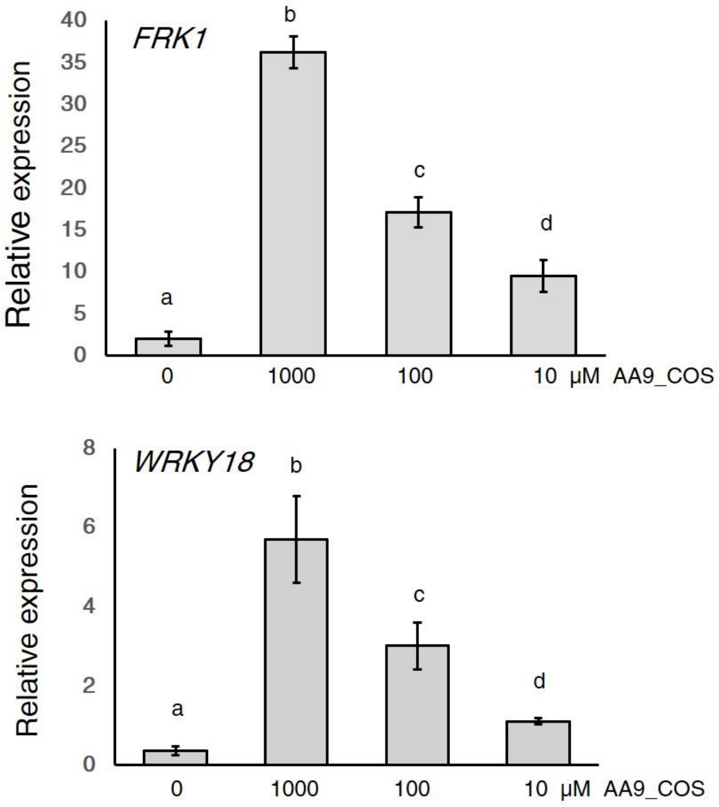


**Supplementary Figure 3: AA9_COS gradient based *FRK1* and *WRKY18* gene expression in Arabidopsis.** The expression of *FRK1* and *WRKY18* genes was evaluated in Col-0 Arabidopsis seedling. Fourteen-day-old seedlings were treated with increasing concentration (10, 100, 1000 µM) of AA9_COS or mock and gene expression was evaluated 1h after treatments. Bars represent the mean ± SD of three independent pools (n = 3) of fifteen seedlings each. Different letters correspond to significantly different expression levels according to one-way ANOVA test ($p < 0.05$).

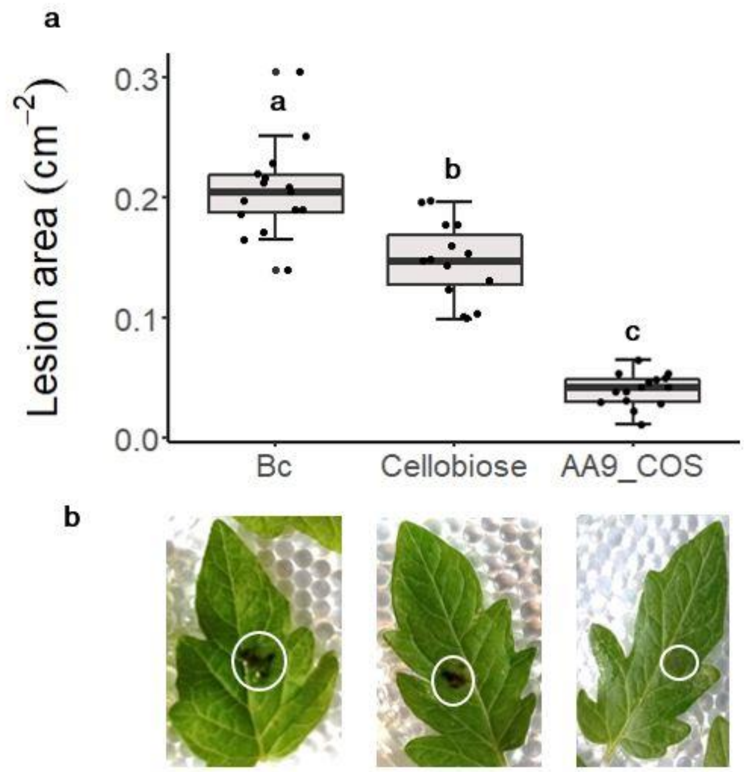


**Supplementary Figure 4: assessment of *B. cinerea* symptoms in tomato plants. a** Two-month old leaves were treated with 100 µM AA9_COS, 100 µM cellobiose or mock and 24 h later, detached leaves were spotted with 10 µl *B. cinerea* spore suspension ($10^5$ spores $mL^{-1}$). Pictures were taken three days after infection and symptomatology was evaluated by ImageJ software. Five leaves from three plants (n = 3) were analyzed for each experiment. The experiments were performed and analysed three independent times. Different letters denote one-way ANOVA test with Tukey's post-hoc multiple comparisons. **b** Representative pictures of tomato leaves are reported.

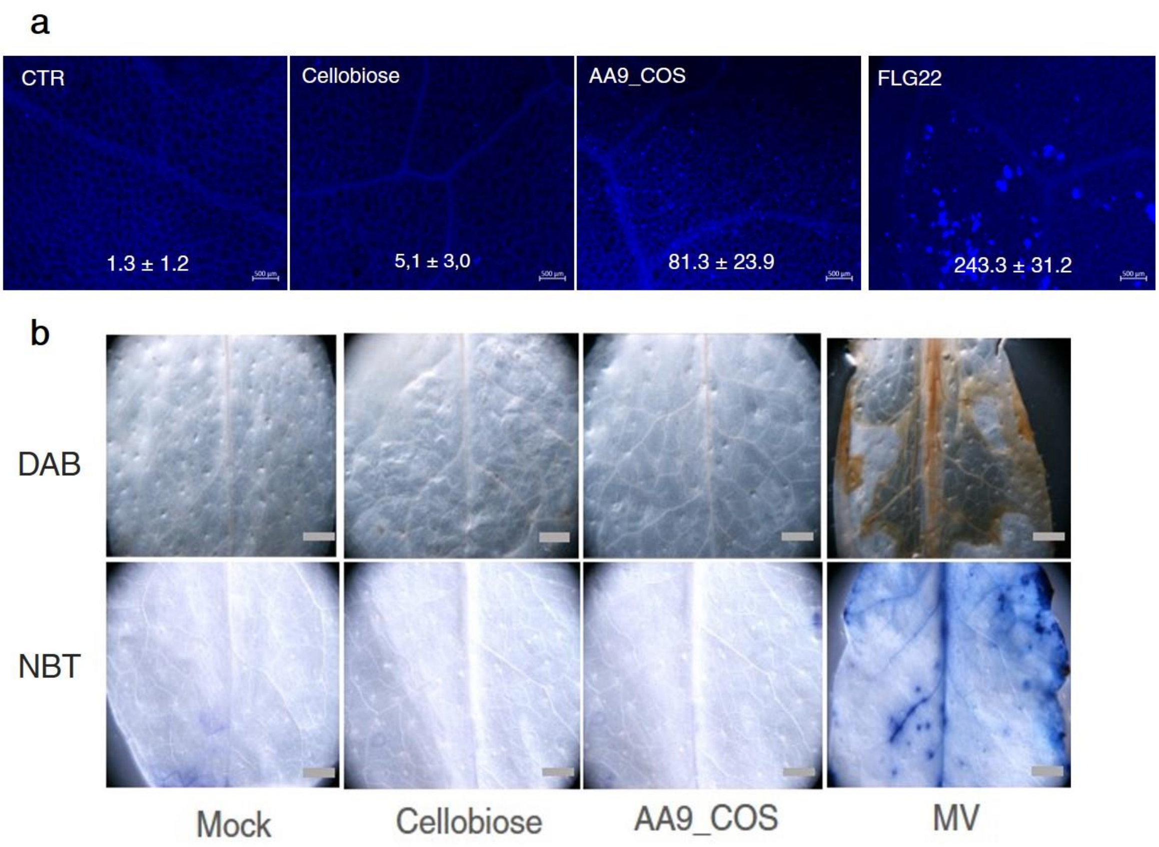


**Supplementary Figure 5. Aniline blue staining for callose detection 6h after treatment and DAB and NBT staining**. **a** Five-week-old Arabidopsis leaves were drop-treated with the indicated compounds and callose was stained 6 h after treatments (for quantification see graph reported in Fig. 2d). Representative pictures are reported. **b** $H_2O_2$ and $O_2^-$ detection was performed in five-week-old Arabidopsis plants 24 h following treatments and representative images of DAB- and NBT-stained leaves are reported.

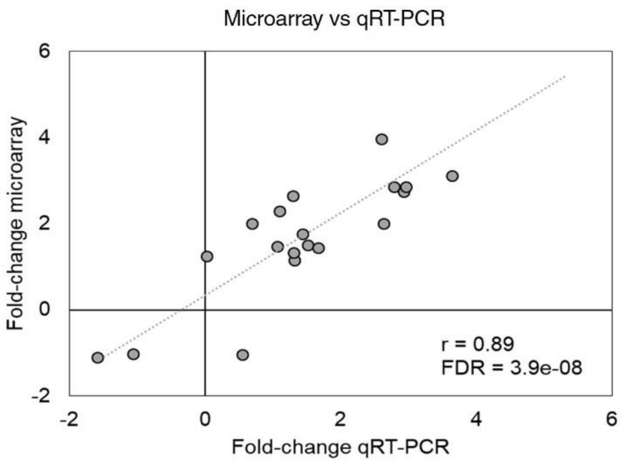


| | qRT-PCR | | Microarray | |
|---|---|---|---|---|
| | AA9_COS | Cellobiose | AA9_COS | Cellobiose |
| **MPK3** | 1.52 | 0.02 | 1.51 | 1.24 |
| **MPK6** | 1.32 | 0.55 | 1.14 | -1.03 |
| **PAD3** | 1.67 | -1.06 | 1.44 | -1.02 |
| **PR1** | 1.44 | 1.29 | 1.77 | 2.65 |
| **LOX4** | 2.78 | 0.69 | 2.86 | 2.00 |
| **WRKY33** | 2.64 | 1.31 | 2.01 | 1.33 |
| **WRKY22** | 2.96 | 1.10 | 2.86 | 2.29 |
| **WRKY18** | 3.65 | 2.60 | 3.12 | 3.96 |
| **IGMT2** | 5.35 | -1.59 | 6.17 | -1.09 |
| **PEN3** | 2.93 | 1.07 | 2.75 | 1.47 |

**Supplementary Figure 6: correlation between microarray-qRT-PCR data.** The fold changes of gene expression determined by qRT-PCR and by cDNA-microarray are plotted for a subset of ten key differentially expressed genes in Arabidopsis. r indicates the correlation and FDR the p-value false discovery rate.

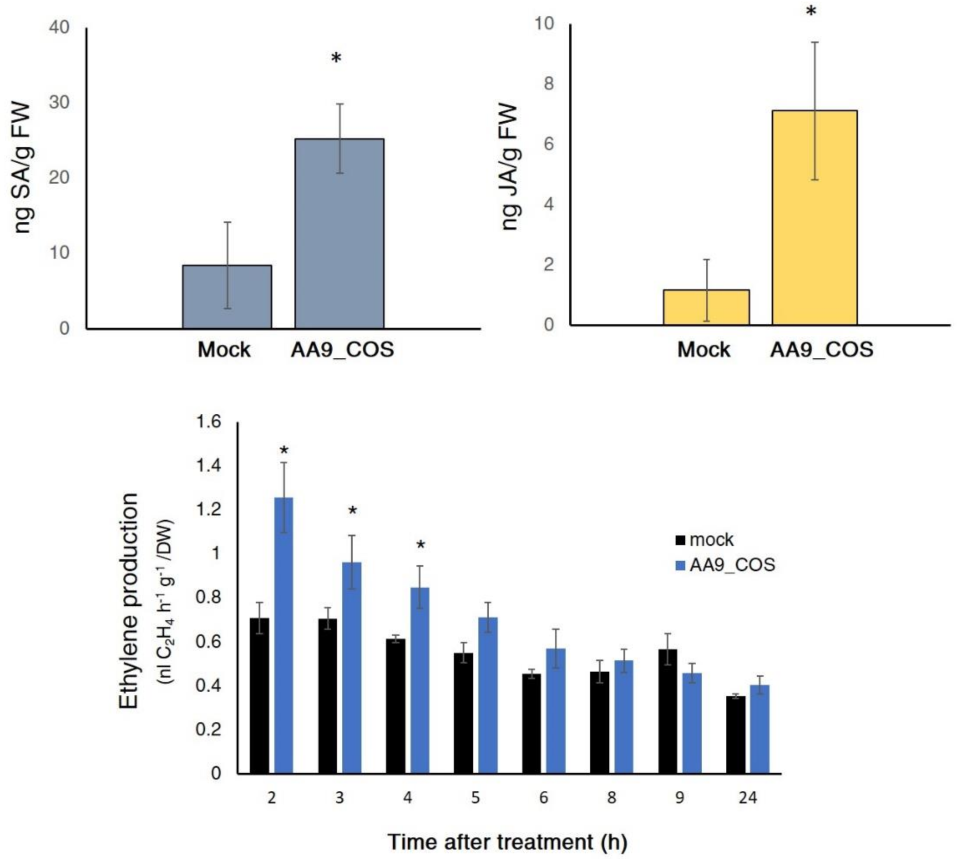


**Supplementary Figure 7: Phytohormones quantification.** Salicylic acid (SA) and jasmonic acid (JA) contents were quantified by high-performance liquid chromatography-electrospray ionization-tandem mass spectrometry (HPLC-ESI-MS/MS). Three pools (n = 3) of fifteen leaves from three five-week-old Arabidopsis plants were treated with 100 µM AA9_COS or mock and leaves were collected, pooled and immediately frozen in liquid nitrogen 24 h after treatments. For each independent pool, JA and SA extraction and quantification was performed. Bars represent mean and standard deviation of three pool, asterixis indicate significant differences (one-way ANOVA test). Ethylene was measured in a time course-analysis using square dishes containing 100 fourteen-day-old seedlings each. Bars represent mean and standard error of eight independent square dishes (n = 8). Asterixis indicate significant differences (one-way ANOVA test). DW: dry weight. FW: Fresh weight.

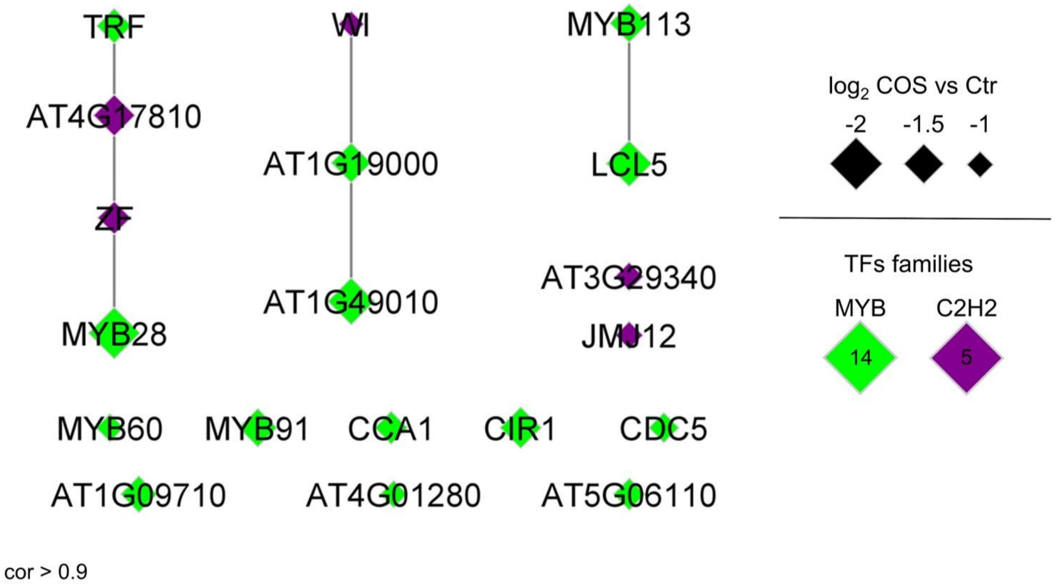


**Supplementary Figure 8: correlation network of AA9_COS-downregulated transcription factors (TFs).** The correlation network was performed using significantly ($p < 0.05$) downregulated genes belonging to TF families. Grey edges indicate a positive correlation among TF genes whereas the rhombus sizes represent the different magnitude of gene expression reported as $\log_2$ FC AA9_COS vs mock. Only co-expressed upregulated TFs with $r > 0.9$ and TF families with at least five members downregulated in AA9_COS were visualized in the network.

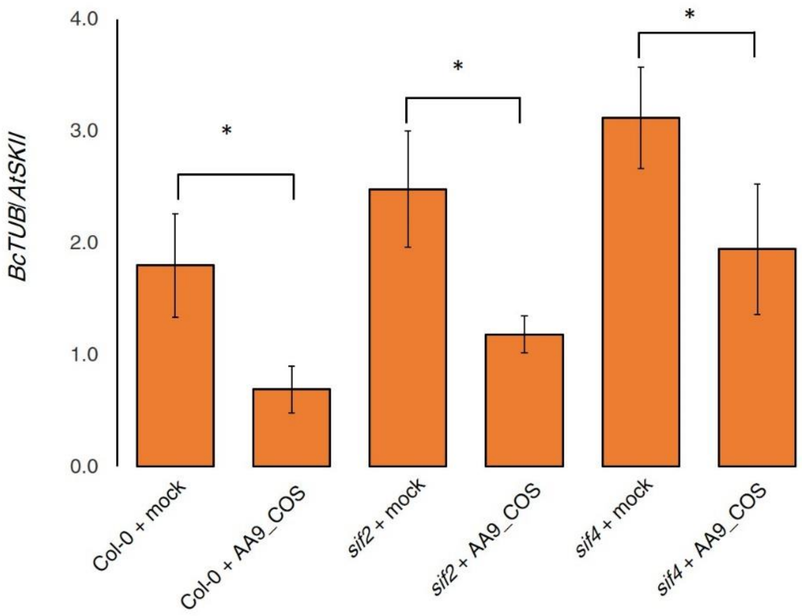


**Supplementary Figure 9: *B. cinerea in-planta* growth in *sif2* and *sif4* loss-of-function lines.** Three independent pools (n = 3) consisting of fifteen leaves from three five-week-old *sif2* and *sif4* plants were treated either with 100 µM AA9_COS or mock and *B. cinerea* inoculation ($10^5$ spore $ml^{-1}$) was carried out 24 h later. The *in-planta* growth of *B. cinerea* was determined three days after infection by qRT-PCR using housekeeping genes specific for Arabidopsis (*AtSKII*) and *B. cinerea* (*BcTUB*). Data represent the mean ± SD from the measurements of the three independent pools. Asterisks denote significant differences according to one-way ANOVA test ($p < 0.05$).

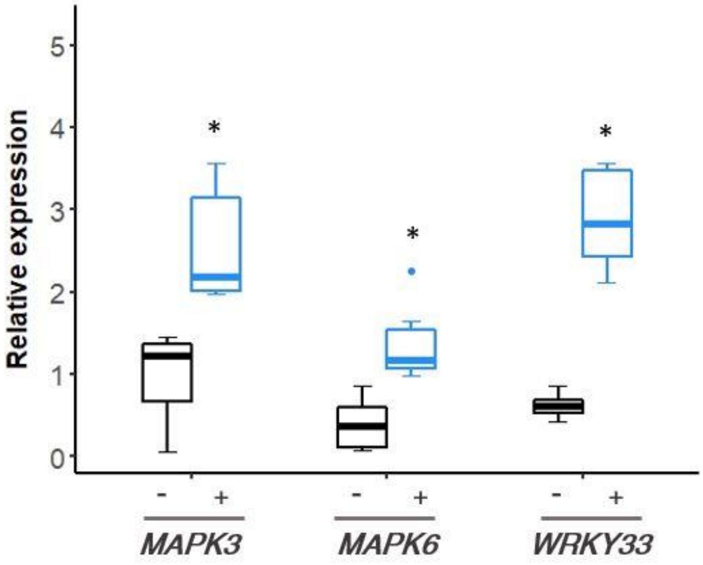


**Supplementary Figure 10: AA9_COS-dependent gene expression analysis of selected genes in five-week-old Arabidopsis plants.** Three independent pools (n = 3), each consisting of fifteen leaves from five-week-old Col-0 plants, were treated either with 100 µM AA9_COS or mock and gene expression was evaluated 1h after treatments. Data represent the mean ± SD from the measurements of the three independent pools. Asterisks indicate significant differences according to one-way ANOVA test ($p < 0.05$) compared to mock-treated plants.

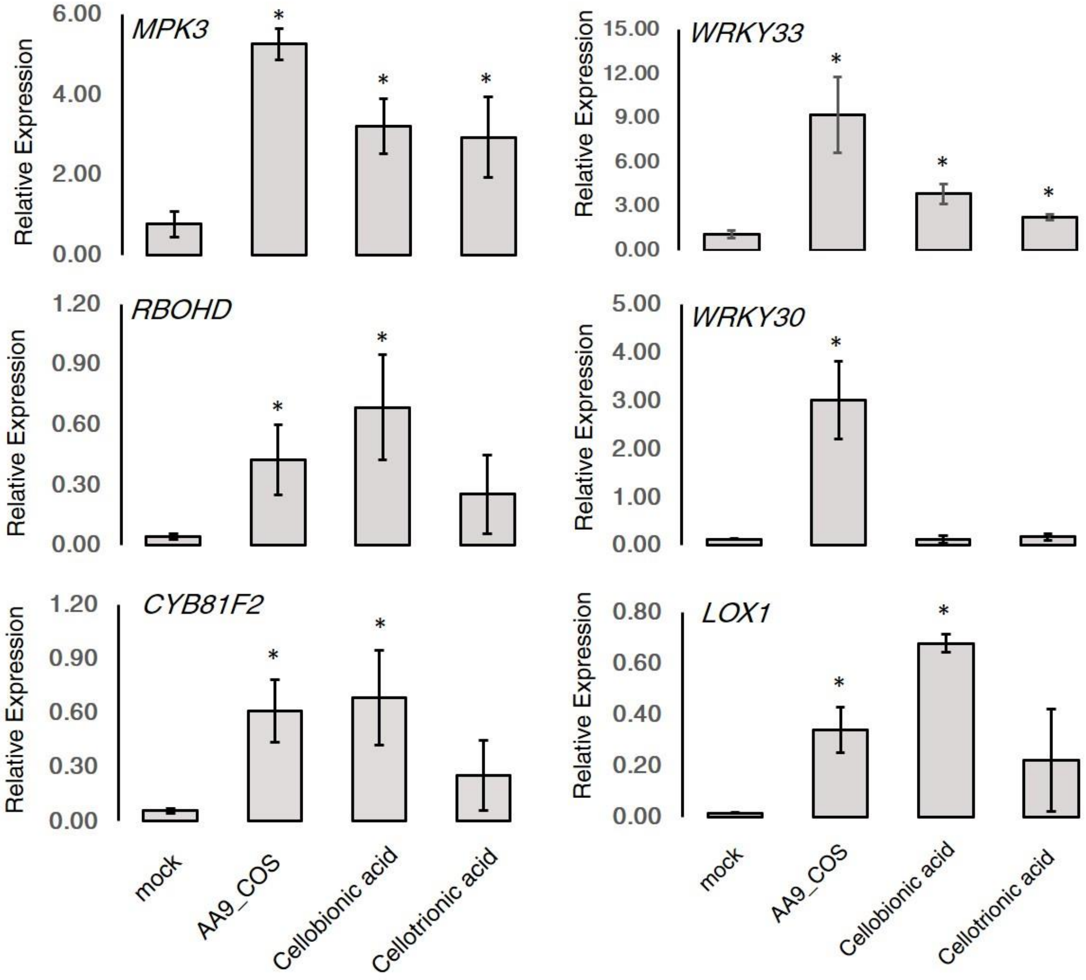


**Supplementary Figure 11: gene expression analysis following cellobionic and cellotrionic acid treatments.** The expression of three marker genes selected from[54] (*CYB81F2*, *RBOHD*, and *WRKY30*) plus *MPK3* and *LOX1* selected from[34] was evaluated in Col-0 Arabidopsis seedlings. Three independent pools (n = 3) of fifteen 14-day-old Arabidopsis seedlings were treated with 100 µM AA9_COS, 100 µM cellobionic acid and 100 µM cellotrionic acid and qRT-PCR were performed 1 h after treatments. Data represent the mean ± SD from the measurements of the three independent pools. Asterisks indicate significant differences compared to mock-treated plants according to one-way ANOVA test ($p < 0.05$). The cellobionic and cellotrionic acids were prepared by oxidation of native cellobiose and cellotriose,

respectively, through Cellobiosedehydrogenase (CDH) obtained from the fungus *Myceliophthora thermophila*.

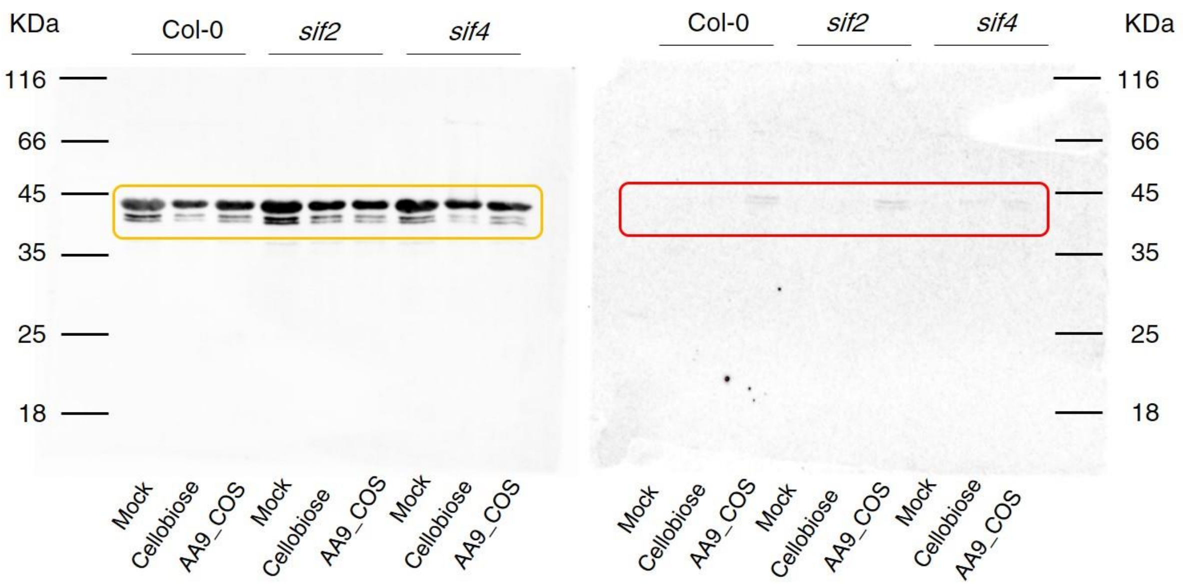


**Supplementary Figure 12: whole western blot images for Figure 7c**

| Transcription factor family | Gene identifier | Probeset | Annotation | Fold change (treatment / mock) | | | |
|---|---|---|---|---|---|---|---|
| | | | | AA9_COS | | Cellobiose | |
| | | | | Expression | p-value | Expression | p-value |
| WRKY TFs | AT5G49520 | 13512671 | WRKY48 | 3.906 | 0.03 | 3.07 | 0.04 |
| | AT4G31800 | 13476200 | WRKY18 | 3.117 | 0.00 | 3.96 | 0.00 |
| | AT4G01250 | 13480486 | WRKY22 | 2.859 | 0.00 | 2.29 | 0.00 |
| | AT5G24110 | 13530150 | WRKY30 | 2.797 | 0.00 | 1.38 | 0.01 |
| | AT2G30250 | 13414799 | WRKY25 | 2.729 | 0.01 | 2.18 | 0.01 |
| | AT5G15130 | 13501728 | WRKY72 | 2.690 | 0.00 | 1.38 | 0.06 |
| | AT4G31550 | 13492012 | WRKY11 | 2.292 | 0.00 | 1.88 | 0.00 |
| | AT4G01720 | 13464517 | WRKY47 | 2.267 | 0.02 | 1.54 | 0.08 |
| | AT4G23550 | 13472446 | WRKY29 | 2.249 | 0.04 | 1.50 | 0.10 |
| | AT1G62300 | 13382260 | WRKY6 | 2.173 | 0.03 | 1.46 | 0.19 |
| | AT2G38470 | 13415587 | WRKY33 | 2.010 | 0.04 | -1.25 | 0.27 |
| | AT1G18860 | 13443480 | WRKY61 | 1.905 | 0.01 | 1.20 | 0.48 |
| | AT1G80840 | 13499405 | WRKY40 | 1.853 | 0.01 | -1.05 | 0.59 |
| | AT5G13080 | 13348179 | WRKY75 | 1.848 | 0.05 | 1.23 | 0.16 |
| | AT2G23320 | 13440324 | WRKY15 | 1.745 | 0.00 | -1.06 | 0.65 |
| | AT2G24570 | 13469960 | WRKY17 | 1.621 | 0.02 | 1.32 | 0.12 |
| | AT2G30590 | 13398985 | WRKY21 | 1.380 | 0.00 | 1.31 | 0.07 |
| NAC TFs | AT3G04070 | 13444373 | NAC47 | 2.775 | 0.00 | 2.03 | 0.01 |
| | AT3G15500 | 13428695 | NAC3 | 2.619 | 0.01 | 1.26 | 0.47 |
| | AT2G43000 | 13420626 | NAC42 | 2.135 | 0.04 | 1.20 | 0.01 |
| | AT5G04410 | 13497454 | NAC2 | 1.587 | 0.00 | 1.34 | 0.00 |
| | AT3G18400 | 13450847 | NAC58 | 1.456 | 0.01 | 1.36 | 0.01 |
| | AT4G28530 | 13490873 | NAC74 | 1.442 | 0.02 | 1.09 | 0.24 |
| | AT5G13180 | 13500896 | NAC83 | 1.352 | 0.03 | 1.23 | 0.05 |
| | AT2G33480 | 13400268 | NAC41 | 1.297 | 0.02 | -1.03 | 0.59 |
| | AT5G18270 | 13527755 | NAC87 | 1.258 | 0.01 | 1.66 | 0.00 |
| ERF TFs | AT1G21910 | 13343195 | ERF012 | 4.159 | 0.00 | 3.47 | 0.01 |
| | AT1G36060 | 13375795 | ERF055 | 3.075 | 0.03 | 1.21 | 0.56 |
| | AT5G61600 | 13542224 | ERF104 | 2.187 | 0.03 | 1.19 | 0.48 |
| | AT5G07580 | 13488463 | ERF106 | 1.638 | 0.02 | 1.38 | 0.04 |
| | AT1G33760 | 13347536 | ERF022 | 1.610 | 0.02 | 1.22 | 0.05 |
| | AT1G77640 | 13360299 | ERF013 | 1.450 | 0.01 | 1.66 | 0.07 |
| | AT3G50260 | 13437652 | ERF011 | 1.447 | 0.01 | -1.03 | 0.74 |
| | AT2G33710 | 13416460 | ERF112 | 1.304 | 0.02 | 1.09 | 0.51 |
| | AT5G65130 | 13519600 | ERF057 | 1.129 | 0.04 | 1.05 | 0.72 |

**Supplementary Table 1. Gene expression of *WRKYs*, *NACs* and *ERFs* TF following AA9_COS and cellobiose treatments.** Fourteen-day-old Arabidopsis seedlings were treated with 100 µM AA9_COS, 100 µM cellobiose and mock and transcriptome analysis was performed 1 h after treatments. Differentially expressed genes belonging to the *WRKY*, *NAC* and *ERF* TF families are reported.

**PTI associted RLKs**

| AGI code | Description | Gene symbol | AA9_COS / mock FC | AA9_COS / mock p-value | Cellobiose / mock FC | Cellobiose / mock p-value |
|---|---|---|---|---|---|---|
| AT1G51820 | Stress induced factor 4 | SIF4 | 17.53 | 0.002 | 8.28 | 0.006 |
| AT2G19190 | FLG22-induced receptor-like kinase 1 | FRK1 | 14.21 | 0.000 | 1.31 | 0.163 |
| AT1G51850 | Stress induced factor 2 | SIF2 | 17.20 | 0.001 | 3.82 | 0.028 |
| AT5G48380 | BAK1-interacting receptor-like kinase | BIR1 | 2.33 | 0.003 | 1.95 | 0.006 |
| AT1G74360 | Nematode-induced LRR-RLK1 | NILR1 | 2.45 | 0.017 | 1.38 | 0.048 |
| AT5G46330 | Flagelline-sensitive 2 | FLS2 | 2.82 | 0.007 | 1.40 | 0.400 |
| AT2G23770 | Lysin motif receptor-like kinase 4 | LYK4 | 1.88 | 0.005 | 1.27 | 0.086 |
| AT2G31880 | Suppressor of BIR1 1 | SOBIR1 | 2.21 | 0.017 | 1.54 | 0.087 |
| AT3G21630 | Chitin elicitor receptor kinase 1 | CERK1 | 1.89 | 0.000 | 1.33 | 0.002 |
| AT4G33430 | BRI1-associated receptor kinase | BAK1 | 1.77 | 0.002 | 1.15 | 0.251 |

**WAKs and WALKs**

| AGI code | Description | Gene symbol | AA9_COS / mock FC | AA9_COS / mock p-value | Cellobiose / mock FC | Cellobiose / mock p-value |
|---|---|---|---|---|---|---|
| AT1G79680 | Wall-associated receptor kinase-like 10 | WAKL10 | 5.01 | 0.00 | 1.83 | 0.01 |
| AT1G16130 | Wall-associated receptor kinase-like 2 | WAKL2 | 4.00 | 0.01 | 1.98 | 0.01 |
| AT1G21240 | Wall-associated receptor kinase 3 | WAK3 | 2.28 | 0.01 | 2.11 | 0.01 |
| AT2G23450 | Wall-associated receptor kinase-like 14 | WAKL14 | 1.71 | 0.01 | 1.26 | 0.11 |

**Cell Wall Damage-associated receptors**

| AGI code | Description | Gene symbol | AA9_COS / mock FC | AA9_COS / mock p-value | Cellobiose / mock FC | Cellobiose / mock p-value |
|---|---|---|---|---|---|---|
| AT5G54380 | Theseus 1 | THE1 | 1.82 | 0.01 | 2.95 | 0.00 |
| AT3G51550 | Feronia | FER | 1.67 | 0.00 | 2.11 | 0.00 |

**Cysteine-rich receptor-like protein kinase**

| AGI code | Description | Gene symbol | AA9_COS / mock FC | AA9_COS / mock p-value | Cellobiose / mock FC | Cellobiose / mock p-value |
|---|---|---|---|---|---|---|
| AT4G23190 | Cysteine-rich receptor-like protein kinase 11 | CRK11 | 4.78 | 0.004 | 2.23 | 0.034 |
| AT4G23220 | Cysteine-rich receptor-like protein kinase 14 | CRK14 | 3.98 | 0.008 | 1.70 | 0.141 |
| AT4G23200 | Cysteine-rich receptor-like protein kinase 12 | CRK12 | 3.53 | 0.001 | 5.37 | 0.002 |
| AT4G23270 | Cysteine-rich receptor-like protein kinase 19 | CRK19 | 2.91 | 0.001 | 1.59 | 0.036 |
| AT4G23280 | Cysteine-rich receptor-like protein kinase 20 | CRK20 | 3.46 | 0.006 | 1.16 | 0.372 |
| AT4G21410 | Cysteine-rich receptor-like protein kinase 29 | CRK29 | 2.35 | 0.004 | 1.39 | 0.245 |
| AT4G23130 | Cysteine-rich receptor-like protein kinase 5 | CRK5 | 2.70 | 0.003 | 1.80 | 0.003 |
| AT4G23180 | Cysteine-rich receptor-like protein kinase 10 | CRK10 | 1.95 | 0.005 | 1.05 | 0.735 |
| AT1G70520 | Cysteine-rich receptor-like protein kinase 2 | CRK2 | 1.74 | 0.005 | 1.34 | 0.098 |

**Sugar transporters**

| AGI code | Description | Gene symbol | AA9_COS / mock FC | AA9_COS / mock p-value | Cellobiose / mock FC | Cellobiose / mock p-value |
|---|---|---|---|---|---|---|
| AT3G19930 | Sugar transport protein 4 | STP4 | 4.11 | 0.000 | 2.31 | 0.003 |
| AT5G26340 | Sugar transport protein 13 | STP13 | 3.04 | 0.018 | 1.28 | 0.448 |
| AT1G11260 | Sugar transport protein 1 | STP1 | 1.34 | 0.012 | 1.13 | 0.331 |
| AT3G28007 | Bidirectional sugar transporter 4 | SWEET4 | 1.58 | 0.018 | -1.08 | 0.240 |
| AT4G15920 | Bidirectional sugar transporter 17 | SWEET17 | -1.59 | 0.026 | 1.04 | 0.824 |
| AT3G14770 | Bidirectional sugar transporter 2 | SWEET2 | -1.23 | 0.306 | -1.14 | 0.416 |
| AT3G16690 | Bidirectional sugar transporter 16 | SWEET16 | -1.06 | 0.633 | 1.77 | 0.002 |
| AT2G02860 | Sucrose transporter 3 | SUT2 | 2.23 | 0.001 | 2.88 | 0.000 |
| At1g71880 | Sucrose-proton symporter 1 | SUC1 | 2.10 | 0.019 | 2.05 | 0.024 |
| AT5G43610 | Sucrose-proton symporter 6 | SUC6 | 1.28 | 0.355 | 1.22 | 0.502 |

**Supplementary Table 2.** Gene expression data for Pattern Recognition Receptors (PRRs), Receptor-Like Kinases (RLKs), Wall associated Kinases (WAKs), Cell Wall Damage associated kinases and sugar transporters induced 1 h after 100 µM AA9_COS and 100 µM cellobiose treatment in fourteen-day-old Arabidopsis plants (data extracted from transcriptome analysis).

| Arabidopsis | | | | *Botrytis cinerea* | | |
|---|---|---|---|---|---|---|
| AGI code | Gene name | Orientation | Primer sequence | Gene name | Orientation | Primer sequence |
| AT2G37620 | *AtACT1* | For | CGCCGACAGAATGAGCAAAG | *BcTub* | For | TTCCATGAAGGAGGT TGAGG |
| | | Rev | TGCTGGAAGGTACTGAGGGA | | Rev | TACCAACGAAGGTGGAGGAC |
| AT5G60390 | *AtEF1α* | For | AGGTCCACCAACCTTGACTG | *BCIN_02g02040* | For | CGGCTGGCTGATACCTAAAGAT |
| | | Rev | CCGTTCCAATACCACCAATC | | Rev | GAAGTTCAACCTTTCCACCTGC |
| AT4G34210 | *AtSK11* | For | CTGGTGAGAGCGGTGTTGAT | *BCIN_03g05890* | For | TACGAGCAGAAGTCATCGCTTT |
| | | Rev | CCCAAGACCTACCCCAAAGC | | Rev | GCCCCTGGGAATAATACTCCAG |
| AT2G43790 | *AtMAPK6* | For | ACCGTAAGCCACTCTTCCCT | *BCIN_05g08230* | For | ACTCATCCAGGACCCATTCAAC |
| | | Rev | GGCGAGGATAAGGTGGAAGC | | Rev | AGCTCATGTTACCAGCATCCAT |
| AT3G45640 | *AtMAPK3* | For | GGTGGAAACTCACGGAGGAC | *BCIN_06g00480* | For | AACAATGCAGGCGTACAGAGTA |
| | | Rev | GCTCGTTCGTCTCCGTATCC | | Rev | TTGGAGTTGAGGTTCCTGTTCC |
| AT3G26830 | *AtPAD3* | For | GGAGTCGCTGGCATAACACT | *BCIN_06g07050* | For | CCCTACTGTTCTCTCTCATGGC |
| | | Rev | GTCCCCAAGTGTTGTCCGAA | | Rev | CGCAACAAACCCCAAATCCAAT |
| AT1G59870 | *AtPEN3* | For | GCTATTGTGATGAGGGCGGTAA | *BCIN_09g06730* | For | CACAACTGTTGACAAGACCACC |
| | | Rev | AGAGTTTTGACCCAATGGACCC | | Rev | CCAAGGACTCTGGGATGGTAAC |
| AT4G31800 | *AtWRKY18* | For | AGGTTACAAGAGACAACCCGTC | *BCIN_09g06750* | For | CTCTCTCAGCTAAAATTGCCGC |
| | | Rev | CAGAAGCATTTGGACCCAAGTG | | Rev | CCACCCTCGTCAATCTTGAAGA |
| AT4G01250 | *AtWRKY22* | For | AAACCATCCGATCAACAGACGA | *BCIN_11g05360* | For | TCCAAGGCAATGGTCCTATCAC |
| | | Rev | ATCATCGCTAACCACCGTATCC | | Rev | CCACAGCGTCCATCTCAAACTA |
| AT5G24110 | *AtWRKY30* | For | CCAAGTTTCTCAGGGTGGAG | *BCIN_12g03920* | For | CCTGGTGGCATGTTACTCTTCT |
| | | Rev | CATCGTCCAGCGTTCTATCA | | Rev | GTTGATGGGAAATGTAGCCGTG |
| AT2G38470 | *AtWRKY33* | For | TGGAGAGAGCATCACACGAC | *BCIN_15g03140* | For | GGCTTCTCAAGGGGGATCTTC |
| | | Rev | GTGCTCTGTTTGTGGCGTAA | | Rev | GTCTTAGTAGCAGACCCAGACC |
| AT5G51830 | *AtFRK1* | For | TGAGCTGGGAAGAGAGGTTGAA | | | |
| | | Rev | TAGATAACCCGAAGTCCGCCAT | | | |
| AT1G21120 | *AtIGMT2* | For | ACTGACTCGTTCCTTTCACCC | | | |
| | | Rev | ATAGACCCTCTCCCCCTTTCC | | | |
| AT5G47910 | *AtRBOHD* | For | GACTGGCATTGTGATGGTTG | | | |
| | | Rev | TACCAAAAGGCGTTGAAACC | | | |
| AT5G57220 | *AtCYP81F2* | For | GTTTTGATTGTTTTGCCACTTG | | | |
| | | Rev | GGTGGTTTCACTAGGTGGAGAT | | | |
| AT1G55020 | *AtLOX1* | For | GCAAGCAGGACACTTCTGTTC | | | |
| | | Rev | AGCGAGTCGTAGACACCTTCA | | | |
| AT1G51850 | *AtSIF2* | For | ATTTCCCGCAGATGGAGAC | | | |
| | | Rev | CTGAATGGCTTGGGTGATG | | | |
| AT1G51820 | *AtSIF4* | For | TAAAAACCGCATCCATAGTCG | | | |
| | | Rev | CTTGATCCCAGCAACATCATT | | | |

**Supplementary Table 3. List of primers used in the current work.** The reverse and forward primer sequences specific for Arabidopsis or *B. cinerea* used in the current work are reported.

## Supplementary references:


34. Locci, F. *et al.* An Arabidopsis berberine bridge enzyme-like protein specifically oxidizes cellulose oligomers and plays a role in immunity. *Plant J.* **98**, 540–554 (2019).

54. Johnson, J. M. *et al.* A poly(A) ribonuclease controls the cellotriose-based interaction between piriformospora indica and its host arabidopsis. *Plant Physiol.* **176**, 2496–2514 (2018).
