# Supplementary material for: LPMO-oxidized cellulose oligosaccharides evoke immunity in Arabidopsis conferring resistance towards necrotrophic fungus B. cinerea
Source: Commun Biol. 2021 Jun 11;4:727. doi: 10.1038/s42003-021-02226-7 (PMC8196058; doi:10.1038/s42003-021-02226-7)
Supplement: Supplementary file 2 — Description of Additional Supplementary Files [file 42003_2021_2226_MOESM2_ESM.pdf]

Description of Additional Supplementary Files

File name: Supplementary Data 1
Description: GO analysis for Biological Process-terms of the up-regulated genes.

File name: Supplementary Data 2
Description: Hierarchical clustering of differentially expressed genes.

File name: Supplementary Data 3
Description: GO analysis on genes featuring each cluster.

File name: Supplementary Data 4
Description: GO enrichment analyses of DEGs following AA9_COS or cellobiose treatment.

File name: Supplementary Data 5
Description: Raw data used to create figures.
