# Supplementary material for: LPMO-oxidized cellulose oligosaccharides evoke immunity in Arabidopsis conferring resistance towards necrotrophic fungus B. cinerea
Source: Commun Biol. 2021 Jun 11;4:727. doi: 10.1038/s42003-021-02226-7 (PMC8196058; doi:10.1038/s42003-021-02226-7)
Supplement: Supplementary file 8 — Reporting Summary [file 42003_2021_2226_MOESM8_ESM.pdf]

nature research

Corresponding author(s): David Cannella

Last updated by author(s): Feb 25, 2021

Reporting Summary

Nature Research wishes to improve the reproducibility of the work that we publish. This form provides structure for consistency and transparency in reporting. For further information on Nature Research policies, see our Editorial Policies and the Editorial Policy Checklist.

Statistics

For all statistical analyses, confirm that the following items are present in the figure legend, table legend, main text, or Methods section.

n/a	Confirmed	
☐	☒	The exact sample size (*n*) for each experimental group/condition, given as a discrete number and unit of measurement
☐	☒	A statement on whether measurements were taken from distinct samples or whether the same sample was measured repeatedly
☐	☒	The statistical test(s) used AND whether they are one- or two-sided<br>*Only common tests should be described solely by name; describe more complex techniques in the Methods section.*
☒	☐	A description of all covariates tested
☒	☐	A description of any assumptions or corrections, such as tests of normality and adjustment for multiple comparisons
☐	☒	A full description of the statistical parameters including central tendency (e.g. means) or other basic estimates (e.g. regression coefficient) AND variation (e.g. standard deviation) or associated estimates of uncertainty (e.g. confidence intervals)
☒	☐	For null hypothesis testing, the test statistic (e.g. F, t, r) with confidence intervals, effect sizes, degrees of freedom and P value noted<br>*Give P values as exact values whenever suitable.*
☒	☐	For Bayesian analysis, information on the choice of priors and Markov chain Monte Carlo settings
☒	☐	For hierarchical and complex designs, identification of the appropriate level for tests and full reporting of outcomes
☒	☐	Estimates of effect sizes (e.g. Cohen's d, Pearson's r), indicating how they were calculated

Our web collection on statistics for biologists contains articles on many of the points above.

Software and code

Policy information about availability of computer code

Data collection	log2 gene level RMA signal intensity was generated by the Applied Biosystems GeneChip Expression Console v1.4 software
Data analysis	Differentially Expressed Genes (DEGs) were selected using two criteria: log2 fold change ≥ 1- or ≤ -1 and Student's t-test p-value ≤ 0.05. Cytoscape was used for network and GO analysis;

For manuscripts utilizing custom algorithms or software that are central to the research but not yet described in published literature, software must be made available to editors and reviewers. We strongly encourage code deposition in a community repository (e.g. GitHub). See the Nature Research guidelines for submitting code & software for further information.

Data

Policy information about availability of data

All manuscripts must include a data availability statement. This statement should provide the following information, where applicable:

- Accession codes, unique identifiers, or web links for publicly available datasets
- A list of figures that have associated raw data
- A description of any restrictions on data availability

Transcriptome data generated in the current study have been submitted to the ArrayExpress repository, accession E-MTAB-10192. All other source data are included in the article as supplementary raw data.

Field-specific reporting

Please select the one below that is the best fit for your research. If you are not sure, read the appropriate sections before making your selection.

☒ Life sciences ☐ Behavioural & social sciences ☐ Ecological, evolutionary & environmental sciences

For a reference copy of the document with all sections, see nature.com/documents/nr-reporting-summary-flat.pdf

Life sciences study design

All studies must disclose on these points even when the disclosure is negative.

Sample size	For experiments carried out with mature Arabidopsis plants (5-week-old), we always used leaves collected from three plants per condition. For transcriptome and qPCR studies carried out with seedlings (14-day-old), a pool of fifteen seedlings were generated per condition.
Data exclusions	no data were excluded from the analysis
Replication	All the experiments were repeated at least three times with similar results
Randomization	Not applicable. The growth conditions of plants and fungi were maintained equal for the entire work as explained in M&M.
Blinding	Microarray, expressomic, and analytical measurements were all conducted in blind using external lab facilities. Whereas experiments linked to phenotyping were not doable in blind.

Reporting for specific materials, systems and methods

We require information from authors about some types of materials, experimental systems and methods used in many studies. Here, indicate whether each material, system or method listed is relevant to your study. If you are not sure if a list item applies to your research, read the appropriate section before selecting a response.

Materials & experimental systems

n/a	Involved in the study	
☐	☒	Antibodies
☒	☐	Eukaryotic cell lines
☒	☐	Palaeontology and archaeology
☒	☐	Animals and other organisms
☒	☐	Human research participants
☒	☐	Clinical data
☒	☐	Dual use research of concern

Methods

n/a	Involved in the study	
☒	☐	ChIP-seq
☒	☐	Flow cytometry
☒	☐	MRI-based neuroimaging

Antibodies

Antibodies used	Primary antibody anti-AtMPK3 and anti-AtMPK6 were bought from Sigma-Aldrich, the primary antibody anti-Phospho-p44/42 MAPK (Thr202/Tyr204) was bought from Cell Signaling Technology (antibody #9101). The secondary antibody anti-Rabbit IgG conjugated with horseradish peroxidase (HRP) enzyme was bought from Promega.
Validation	All the primary antibodies were selected from relevant scientific publication related to the same species (Arabidopsis thaliana) used in the current work.<br>For example see:<br>- de Azevedo Souza, C. et al. Cellulose-derived oligomers act as damage-associated molecular patterns and trigger defense-like responses. Plant Physiol. 173, 2383–2398 (2017).<br>- Chung, H. S. & Sheen, J. MAPK assays in Arabidopsis MAMP-PRR signal transduction. Methods Mol. Biol. 1578, 155–166 (2017).
